# Cuproptosis of endothelial cells in a hypoxic environment: from molecular classification to PMAIP1-targeted intervention

**DOI:** 10.1186/s41065-026-00667-w

**Published:** 2026-03-19

**Authors:** Jie Wang, Xueying Chen, Huabin Wang, Wenjing Feng, Nan Lin, Xueyun Ren, Manman Wang, Lijun Gan

**Affiliations:** 1 Department of Cardiology, Shandong Provincial Key Medical and Health Discipline of Cardiology, Shandong Provincial Key Medical and Health laboratory of Diagnosis and Treatment of Cardiovascular Diseases, Affiliated Hospital of Jining Medical University, Jining, 272000 China; 2 Jining Key Laboratory of Precise Therapeutic Research of Coronary Intervention, Affiliated Hospital of Jining Medical University, Jining, 272000 China; 3 Department of Pediatrics, Affiliated Hospital of Jining Medical University, Jining Medical University, Jining, 272000 China; 4 Jining Key Laboratory for Prevention and Treatment of Severe Infection in Children, Affiliated Hospital of Jining Medical University, Jining, 272000 China; 5 Shandong Provincial Key Medical and Health Discipline of Pediatric Internal Medicine, Affiliated Hospital of Jining Medical University, Jining, 272000 China; 6 Shandong University of Traditional Chinese Medicine, Jinan, 250000 China

**Keywords:** Acute myocardial infarction, Cuproptosis, Bioinformatics, Machine learning, PMAIP1

## Abstract

**Background:**

Acute myocardial infarction (AMI) is a critical and fatal cardiovascular condition. The role of cuproptosis as an emerging mechanism in the pathogenesis of AMI remains to be fully elucidated.

**Methods:**

Patient data were acquired from the Gene Expression Omnibus database. Differential expression analysis was performed on cuproptosis-related genes (CRGs). LASSO regression was employed to identify key CRGs and develop a diagnostic classification model for AMI. Weighted gene co-expression network analysis was performed to investigate key modules associated with the disease and its molecular classification. Subsequently, machine learning techniques were used to identify critical genes within these modules. The diagnostic efficacy of these genes for AMI was assessed using receiver operating characteristic analysis. The functions of the identified key genes were ultimately validated at the cellular level.

**Results:**

Six characteristic CRGs were selected via LASSO regression. The AMI diagnostic classification model based on these CRGs demonstrated superior performance. Patients were classified into two subtypes related to cuproptosis, revealing significant differences in enrichment pathways between these subtypes. SYTL3, SCML1, and PMAIP1 were identified as key genes for AMI, with area under the curves of 0.813, 0.795, and 0.873, respectively. Knocking down PMAIP1 expression reduced intracellular copper levels in hypoxia-induced HUVECs.

**Conclusions:**

This study clarifies the role of cuproptosis in AMI and highlights the potential involvement of PMAIP1, providing a theoretical basis for further investigation into cuproptosis in AMI.

**Supplementary Information:**

The online version contains supplementary material available at 10.1186/s41065-026-00667-w.

## Introduction

Coronary artery disease (CAD) is a complex condition characterized by lipid deposition in the inner lining of coronary arteries, leading to blood flow obstruction [1]. Acute myocardial infarction (AMI), a specific manifestation of CAD, results in the death of heart muscle cells due to prolonged coronary ischemia. AMI is characterized by a rapid onset, rapid disease progression, and high mortality rates [2]. Despite advancements in pharmacological treatments and revascularization techniques, AMI remains among the leading causes of death among adults [3]. Traditional cardiovascular risk factors, such as hypertension, diabetes, and smoking, along with markers of myocardial injury, such as troponin and myoglobin, play roles in the diagnosis and prognosis of AMI. However, their limited specificity and sensitivity hinder their ability to fully predict the severity of the condition [4]. Therefore, the search for more sensitive biomarkers to elucidate the initial disturbances and underlying mechanisms of AMI is an urgent focus of current research [5].

The onset of AMI often results in structural and functional damage to the heart, ultimately triggering irreversible cell death through multiple interconnected pathways. Mitochondria are central to the survival and death of cardiac cells, given their critical roles in energy supply, oxidative stress regulation, calcium homeostasis, and inflammation [6, 7]. Recently, Tsvetkov et al. [8] described a novel form of regulated cell death termed “cuproptosis”. This copper-dependent pathway is mechanistically distinct from apoptosis, necroptosis, and ferroptosis. Cuproptosis is mediated by the ferredoxin 1/lipoic acid synthase axis, wherein excess copper ions directly bind to lipoylated proteins of the tricarboxylic acid (TCA) cycle, particularly dihydrolipoamide S-acetyltransferase. This interaction promotes protein aggregation, depletion of iron–sulfur cluster proteins, and subsequent proteotoxic stress [9]. The resulting mitochondrial dysfunction is characterized by disrupted oxidative phosphorylation, elevated expression of heat shock protein 70, and excessive generation of reactive oxygen species (ROS), culminating in cell death [10, 11].

Dysregulated copper homeostasis is increasingly recognized as a key contributor to cardiovascular disease. Clinical studies have reported significantly elevated serum copper concentrations in patients with AMI compared to healthy controls [12, 13]. Mechanistically, copper overload promotes atherosclerosis via multiple pathways. As a redox-active metal, excess copper catalyzes Fenton-like reactions with hydrogen peroxide to generate highly reactive hydroxyl radicals, inducing lipid peroxidation, DNA strand breaks, and impairing antioxidant enzyme activity, thereby exacerbating oxidative stress. Furthermore, copper overload disrupts endothelial function by altering nitric oxide (NO) homeostasis; elevated copper levels upregulate inducible nitric oxide synthase (iNOS), resulting in excessive NO production and peroxynitrite formation [14]. In addition, copper excess stimulates inflammation by promoting the release of pro-inflammatory cytokines within the arterial wall. Specifically, Cu²⁺ enhances interleukin (IL)-6 secretion and activates MAP kinases in cardiac cells, while copper-induced ROS further activate nuclear factor-κB (NF-κB), a master regulator of inflammatory gene expression [15]. Nevertheless, the precise regulatory mechanisms of cuproptosis in endothelial cells during AMI—particularly the molecular determinants of sensitivity to copper-induced cell death—remain poorly defined.

Vascular endothelial dysfunction, an early hallmark of atherosclerosis, plays a pivotal role in AMI pathogenesis [16]. Endothelial cells are particularly vulnerable to metabolic stress due to their high mitochondrial activity and essential role in maintaining vascular homeostasis [14]. Recent studies suggest that endothelial cells exhibit a unique susceptibility to cuproptosis. For instance, copper oxide nanoparticles have been shown to induce cuproptosis in endothelial cells by disrupting the TCA cycle, thereby impairing angiogenesis and promoting vascular dysfunction [10]. These findings highlight the vulnerability of endothelial cells to copper-mediated cytotoxicity, which may contribute to AMI-associated endothelial dysfunction.

Despite growing interest in cuproptosis, its specific role in AMI-related endothelial dysfunction remains incompletely understood. While cuproptosis has been studied extensively in cancer and metabolic diseases, its regulatory mechanisms in cardiac endothelial cells under ischemic conditions remain largely unexplored [17]. Meanwhile, the molecular determinants that distinguish the sensitivity of endothelial cells from other cardiac cell types to cuproptosis have yet to be elucidated [18]. Building upon this foundation, our study establishes three distinct research objectives. First, to identify characteristic cuproptosis-related genes (CRGs) in AMI and develop a comprehensive diagnostic classification model through systematic bioinformatics analysis. Second, to identify key diagnostic genes using weighted gene co-expression network analysis (WGCNA) combined with machine learning approaches. Third, to experimentally validate the functional roles of these key genes in hypoxia-induced cuproptosis within endothelial cells through in vitro studies. This comprehensive investigation seeks to elucidate AMI pathogenesis from the novel perspective of cuproptosis mechanisms. Ultimately, our work aims to provide innovative molecular targets and robust theoretical foundations for cardiovascular disease diagnosis and therapeutic intervention in the precision medicine era.

## Methods

### Data download and data preprocessing

The transcriptome dataset GSE66360, comprising circulating endothelial cells from AMI patients, was retrieved from the GEO database [19]. We downloaded the series matrix file and the corresponding GPL570 annotation file (HG-U133_Plus_2, Affymetrix Human Genome U133 Plus 2.0 Array). This dataset encompasses transcriptome data from 49 AMI patients and 50 healthy controls. Data preprocessing was performed using the limma package in R software [20]. Multiple probes mapping to identical genes were consolidated using the avereps function, which calculates the mean expression value as the representative gene expression level. Between-array quantile normalization was implemented using the normalizeBetweenArrays function to ensure data comparability across samples. Gene annotation was executed using Perl v5.30.0, ultimately generating a normalized expression matrix for downstream analyses.

### Differential expression and correlation analysis of CRGs

Based on previous literature, mRNA expression levels of 19 CRGs were extracted from all samples [8, 21]. CRGs undetectable by microarray probes were excluded. Differential expression analysis of CRGs between the AMI and control groups was performed using the Wilcoxon rank-sum test, with the false discovery rate (FDR) controlled by the Benjamini, Krieger and Yekutieli (BKY) two-stage step-up method. Heatmaps and boxplots were generated using pheatmap and ggboxplot packages. For differentially expressed CRGs identified through multiple testing correction, Spearman correlation analysis evaluated inter-gene relationships, calculating correlation coefficients and significance levels. Correlation visualizations were created using the ggplot2 package.

### Construction of the CRGs AMI diagnostic classification model

LASSO regression was employed to identify characteristic CRGs from differentially expressed genes [22]. Analysis was conducted using the glmnet package in R software. We implemented a 10-fold cross-validation strategy to determine the optimal regularization parameter λ, utilizing deviance as the model performance evaluation metric. Cross-validation was executed via the cv.glmnet function with alpha parameter set to 1. During cross-validation, the λ value that minimized cross-validation deviance was selected as the optimal parameter for final feature gene set determination. CRGs were transformed into binary variables based on median expression levels. A nomogram diagnostic classification model for AMI was constructed using the rms package. Model discriminative performance was assessed through receiver operating characteristic (ROC) curve analysis, while calibration plots evaluated model consistency. Clinical decision curve analysis was performed to assess the clinical utility of the diagnostic classification model.

### Functional enrichment analysis of CRG molecular subtypes and intersubtypes in AMI patients

Consensus clustering and molecular subtype identification were performed on the GSE66360 dataset using differentially expressed CRGs. The ConsensusClusterPlus package [23] was utilized to partition AMI samples into k subtypes via k-means clustering (k = 2–6), employing Euclidean distance as the inter-sample distance metric. The clustering procedure was repeated 1000 times with 80% sample subsampling in each iteration. Optimal cluster number determination was based on consensus matrix heatmap clarity, cumulative distribution function (CDF) curve clustering scores, and relative changes in area under the CDF curves. Principal component analysis (PCA) plots were generated to validate the robustness of the consensus clustering results. Functional enrichment analyses (GO and KEGG) were executed using the clusterProfiler package [24], with *p* < 0.05 defining the significance threshold for enrichment. Gene set enrichment analysis (GSEA) was performed on the gene expression matrix using the “c2.cp.v7.2.symbols.gmt” reference gene set. A FDR < 0.25 and *p*.adjust < 0.05 were considered indicative of significant enrichment. The GSVA package [25] was used for gene set variation analysis, with “c2.cp.kegg.symbols” as the reference gene set, and differential pathway screening was conducted using the limma package with a threshold of *p* < 0.05.

### WGCNA and protein-protein interaction (PPI) analysis

WGCNA was performed using the WGCNA package [26] for both disease-related and typing analyses. A co-expression network was constructed using the WGCNA algorithm. An appropriate soft threshold was chosen to ensure that the constructed network adhered to the scale-free network standard. A topological overlap matrix was established based on the adjacency matrix, followed by hierarchical clustering to generate a clustering tree of genes. A pruning algorithm was applied to define gene modules. The correlation between the obtained modules and disease or typing was analyzed to identify modules with the strongest correlation. Interacting genes from the disease and typing WGCNA analyses were integrated, and the Search Tool for the Retrieval of Interacting Genes (STRING, http://www.string-db.org/) was employed to construct a PPI network, which was visualized using the igraph package [27].

### Use of machine learning methods to screen key genes of disease

Random Forest (RF), Support Vector Machines (SVM), and Generalized Linear Model (GLM) methods were employed to identify key genes associated with the disease [23, 28]. Prior to model training, the dataset was randomly partitioned into training (70%) and testing (30%) sets. To ensure robust model performance and prevent overfitting, all models were trained using 5-fold repeated cross-validation. Model performance was evaluated on the test set, and the most suitable screening method was determined by comprehensive comparison of boxplots of residuals, reverse cumulative distribution diagrams of residuals, and ROC curve analysis. Gene importance scores were calculated using the DALEX package, with the three highest-scoring genes designated as key diagnostic genes for this condition.

RF exhibits superior performance on small-to-medium datasets through ensemble learning, effectively mitigating overfitting risks while providing intrinsic feature importance metrics essential for gene prioritization. SVM demonstrates exceptional proficiency in high-dimensional data processing. The kernel-based SVM framework enables both linear and nonlinear pattern recognition, with its structural risk minimization principle ensuring robust generalization performance on limited sample sizes. GLM offers interpretable coefficients with explicit biological relevance and computational efficiency. The strategic advantages of employing these algorithms in our study include: (1) optimization for small sample datasets, contrasting with deep learning approaches that require thousands of samples; (2) preservation of interpretability essential for biomedical research applications; (3) demonstrated stability compared to gradient boosting methods that may exhibit overfitting on constrained datasets. The integration of RF (ensemble learning), SVM (kernel-based methods), and GLM (linear modeling) encompasses distinct algorithmic paradigms, ensuring comprehensive evaluation while minimizing method-specific biases. This multi-algorithm approach provides robust validation of gene selection results across different computational frameworks.

### Analysis of key gene interaction network and diagnostic ability

The transcription factor network and small molecule compound action network of the key genes were further analyzed using NetworkAnalyst [29] (https://www.networkanalyst.ca/). The modules for transcription factor-gene interactions and protein-chemical interactions were examined, referencing the ENCODE database for transcription factors and protein-chemical interactions. The resulting networks were visualized using Cytoscape software. Differential expression analysis of the key genes was performed using the limma package and presented in boxplot form. ROC curves were employed to evaluate the predictive accuracy of the key genes, with an area under the curve (AUC) greater than 0.7 indicating good predictive ability.

### Cell culture and hypoxia

Human umbilical vein endothelial cells (HUVECs) were obtained from ATCC. The HUVECs were cultured in Dulbecco’s modified Eagle’s medium (DMEM) supplemented with 1% penicillin-streptomycin and 10% fetal bovine serum (FBS) in a 37 °C incubator with 5% CO_2_ in saturated humidity. To establish the cellular hypoxic model, logarithmic-phase HUVECs were cultured in a tri-gas incubator (Thermo Heracell VIOS 160i) under controlled atmospheric conditions (37 °C, 1% O₂, 5% CO₂, 94% N₂) for 48 h with continuous oxygen concentration monitoring. Following hypoxic exposure, cells were harvested for HIF-1α qPCR analysis to verify successful hypoxic induction. For cell culture experiments, cells from different passages were used to ensure reproducibility, ranging from passage 3 to 6.

### siRNA transfection and grouping

A total of 2 × 10^6^ cells per well were seeded into six-well plates and starved for 24 h prior to transfection. For PMAIP1-siRNA transfection, HUVECs were transfected using Lipofectamine 3000 (Thermo Fisher) according to the manufacturer’s protocol. The cells were divided into four groups: control (Control), model (Hypoxia), experimental (si-negative control (NC) + Hypoxia), and si-PMAIP1 + Hypoxia. Target sequences for PMAIP1 siRNA were designed and purchased from Genepharma, with the interference sequences listed in Supplemental Table 1.

### Measurement of cell copper (Cu) levels

For copper measurement, 150 µL of double-distilled water was added to aliquots of 2 × 10^6^ cells. The homogenate was centrifuged, and the supernatant was retained for protein concentration determination. The relative concentration of Cu²⁺ was detected using a Cell Copper Colorimetric Assay Kit (Elabscience, Wuhan, China), following the manufacturer’s instructions.

### RT-qPCR

Total RNA was extracted from HUVECs using TRIzol reagent (Invitrogen) according to the manufacturer’s instructions. The total RNA was reverse transcribed to cDNA using the ABScript II cDNA First-Strand Synthesis Kit (ABclonal, RK20400). Universal SYBR Green Fast qPCR Mix (ABclonal, RK21203) was used for the amplification of target genes, and the PCR results were analyzed using the delta CT method (2^−ΔΔCT^). The mRNA expression levels of target genes were normalized to β-actin gene expression. The primers used are shown in Supplemental Table 2. Primer specificity was verified by melting curve analysis. All RT-qPCR assays were conducted in triplicate using three independent biological replicates.

### Statistical analysis

All experiments were performed with at least three independent biological replicates. Data are presented as the mean ± standard error of the mean (SEM). Statistical analyses were conducted using R software version 4.1.0 and GraphPad Prism version 9.0. For continuous variables, the Student’s t-test was used to compare differences between normally distributed data, while the Mann-Whitney U test was applied to non-normally distributed data. For group comparisons, one-way ANOVA was applied to normally distributed continuous variables, while the Kruskal–Wallis test was used for non-normally distributed data. Multiple comparisons were further evaluated using Tukey’s post hoc test or Dunn’s post hoc test, as appropriate. All *p*-values were derived from two-tailed tests, and *p* < 0.05 was considered statistically significant.

## Results

### Differential expression and correlation analysis of CRGs

**Fig. 1:**
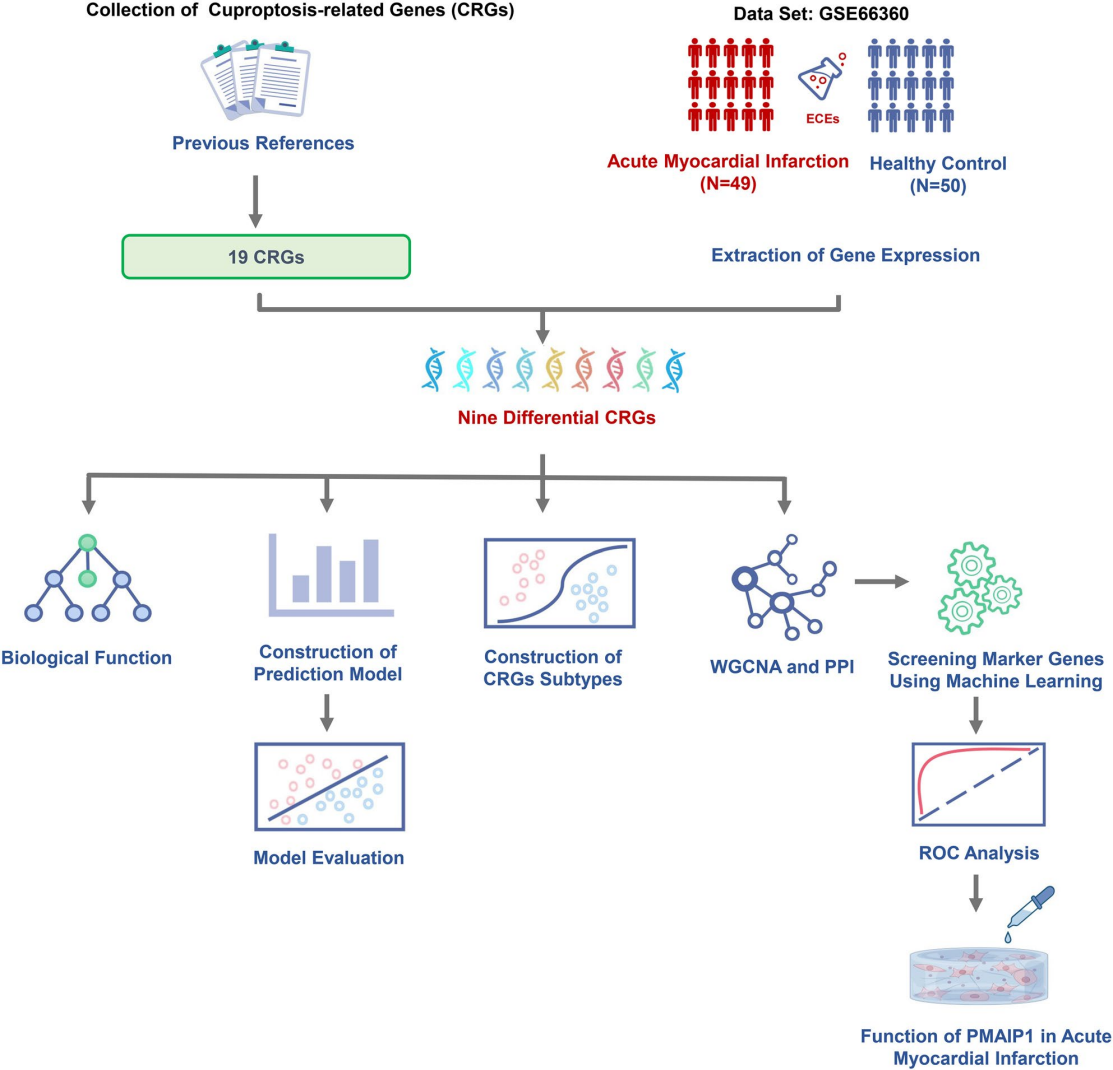
Flowchart illustrating the study design and analysis workflow. PPI, protein-protein interaction; ROC, receiver operating characteristic; WGCNA, weighted gene co-expression network analysis

**Fig. 2:**
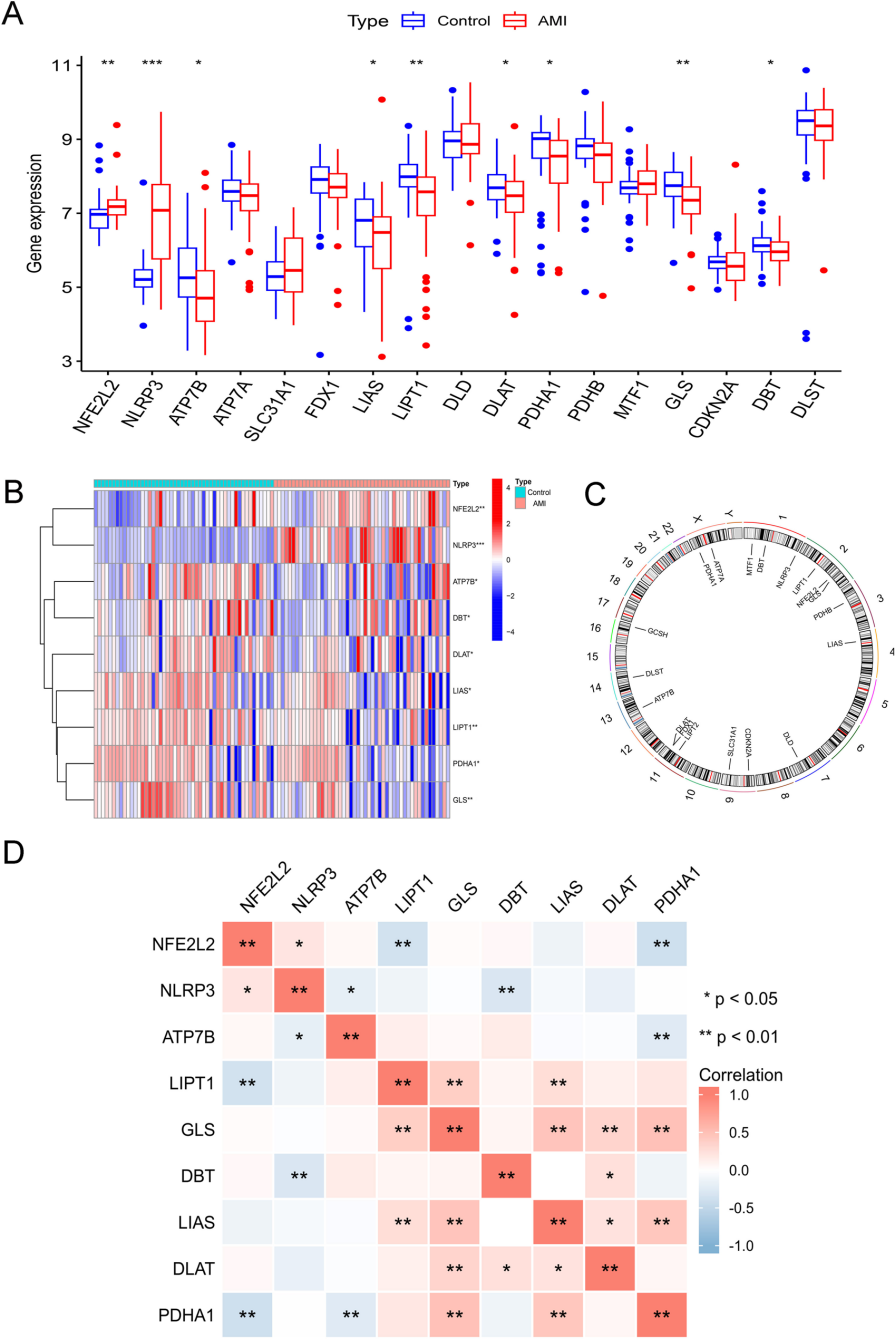
Differential expression and correlation analysis of cuproptosis-related genes (CRGs). (**A**) Boxplot showing the distribution of gene expression levels; (**B**) Heatmap depicting expression patterns of CRGs; (**C**) Chromosomal locations of genes associated with cuproptosis; (**D**) Correlation analysis of differentially expressed CRGs. ^*^p < 0.05, ^**^p < 0.01, ^***^p < 0.001. AMI, acute myocardial infarction

The process for analyzing the dataset is depicted in Fig. 1. By comparing the AMI group with the normal control group, we identified nine differentially expressed CRGs, namely NFE2L2, NLRP3, ATP7B, LIPT1, GLS, DBT, LIAS, DLAT, and PDHA1. Among these, the expression of NFE2L2 and NLRP3 was upregulated in the AMI group, while the other seven CRGs showed downregulated expression (Fig. 2A, B). Chromosomal location information for all CRGs is shown in Fig. 2C. Correlation analysis revealed strong relationships between certain CRGs. Specifically, GLS and LIAS were positively correlated with four other CRGs, while PDHA1 was negatively correlated with NFE2L2 and ATP7B, but positively correlated with GLS and LIAS (Fig. 2D).

### Construction of AMI diagnostic classification model using CRGs

**Fig. 3:**
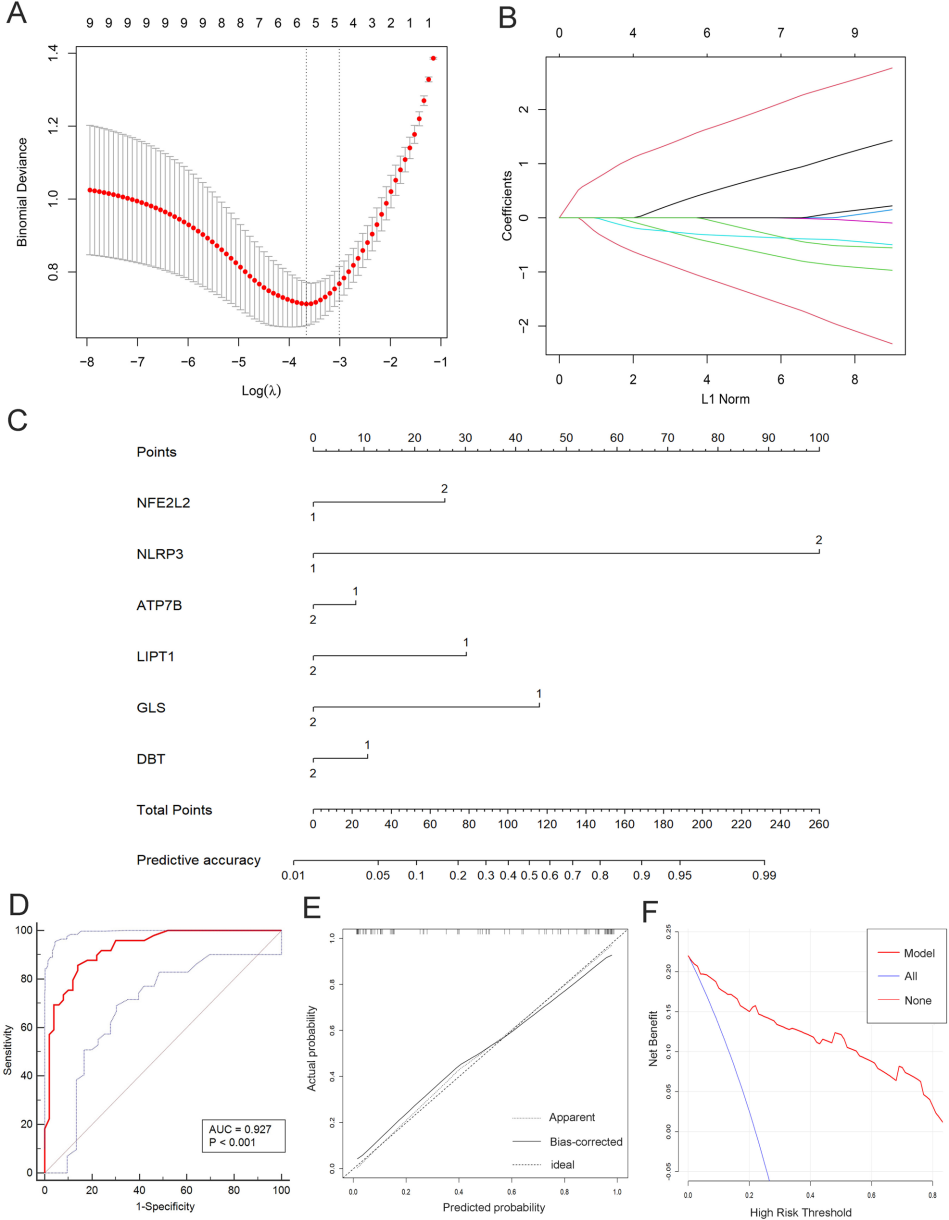
Identification of characteristic cuproptosis-related genes (CRGs) in acute myocardial infarction and construction of a diagnostic classification model. (**A**-**B**) LASSO regression for identifying characteristic CRGs; (**C**) Nomogram of the diagnostic classification model; (**D**) Receiver operating characteristic curve illustrating model performance; (**E**) Calibration curve demonstrating model accuracy; (**F**) Clinical decision curve analysis

The LASSO regression identified characteristic CRGs based on the genes with the smallest cross-validation error. These six CRGs included NFE2L2, NLRP3, ATP7B, LIPT1, GLS, and DBT (Fig. 3A, B). Based on these six genes, we developed the AMI nomogram diagnostic classification model (Fig. 3C). The model demonstrated excellent predictive performance with an AUC value of 0.927. Calibration curves showed good agreement between the predicted and actual outcomes, and clinical decision curves revealed that the net benefit of the diagnostic classification model exceeded that of extreme curves.

### Construction of AMI molecular subtypes and functional enrichment analysis between subtypes

**Fig. 4:**
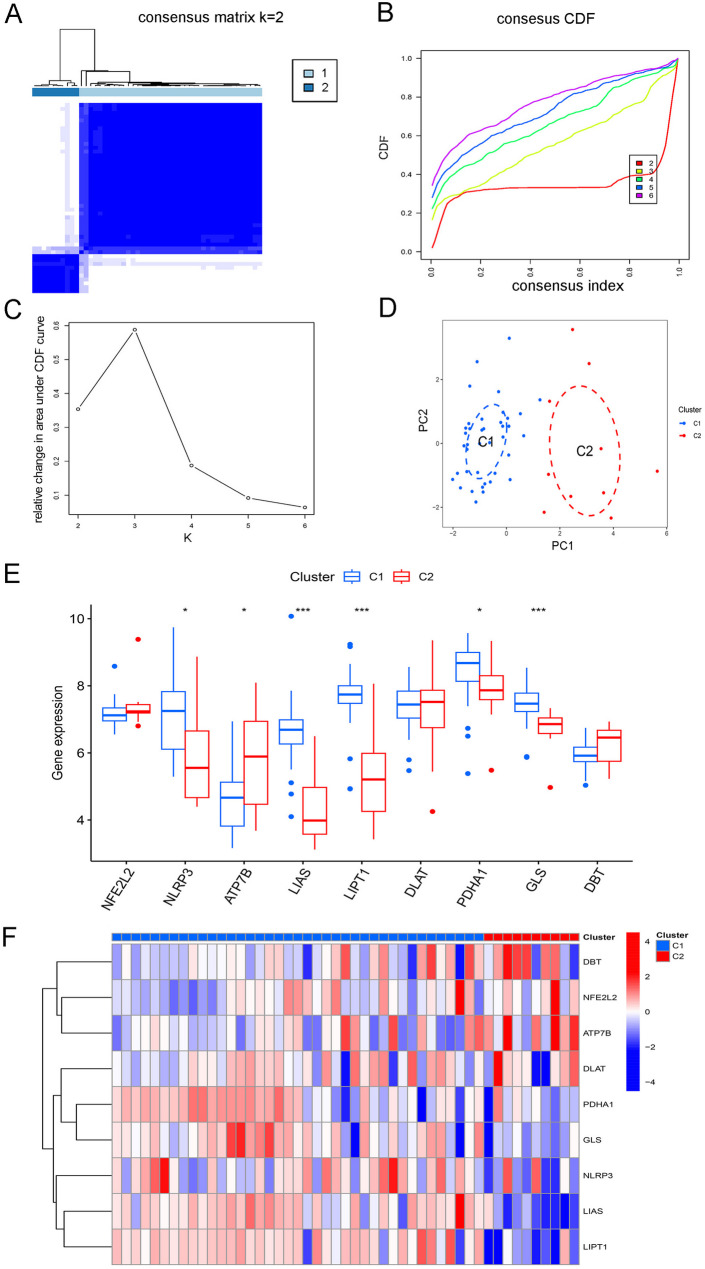
Molecular subtypes in acute myocardial infarction. (**A**) Consensus matrix for k = 2; (**B**) Cumulative distribution function (CDF) curves for k = 2–6; (**C**) Relative change in the area under the CDF curves for k = 2–6; (**D**) Principal component analysis of the two clusters; (**E**) Differences in cuproptosis-related genes (CRGs) between subtypes; (**F**) Heatmap of differentially expressed CRGs. ^*^p < 0.05, ^***^p < 0.001

The analysis selected a dimer solution (k = 2) after evaluating the classification matrix, cluster scores from the CDF curve, and relative changes in the area of the CDF curve; the PCA cluster plot verified that AMI samples could be classified into two subtypes (C1 and C2) (Fig. 4A-D). Six CRGs were differentially expressed between the two subtypes (Fig. 4E, F). Functional enrichment analysis of the DEGs between the subtypes was conducted (Supplemental Fig. 1). GO analysis showed that the DEGs were enriched in processes such as heart morphogenesis, epithelial cell development, and fibrillar collagen trimer. KEGG analysis highlighted pathways such as the AMPK signaling pathway, apoptosis, and PI3K-Akt signaling pathway. GSEA revealed pathways associated with cellular responses to external stimuli, the cell cycle, and the M phase, while GSVA highlighted pathways related to phosphatidylinositol signaling, apoptosis, xenobiotic metabolism by cytochrome P450, and the hedgehog signaling pathway.

### Disease WGCNA, typing WGCNA, and PPI analysis

**Fig. 5:**
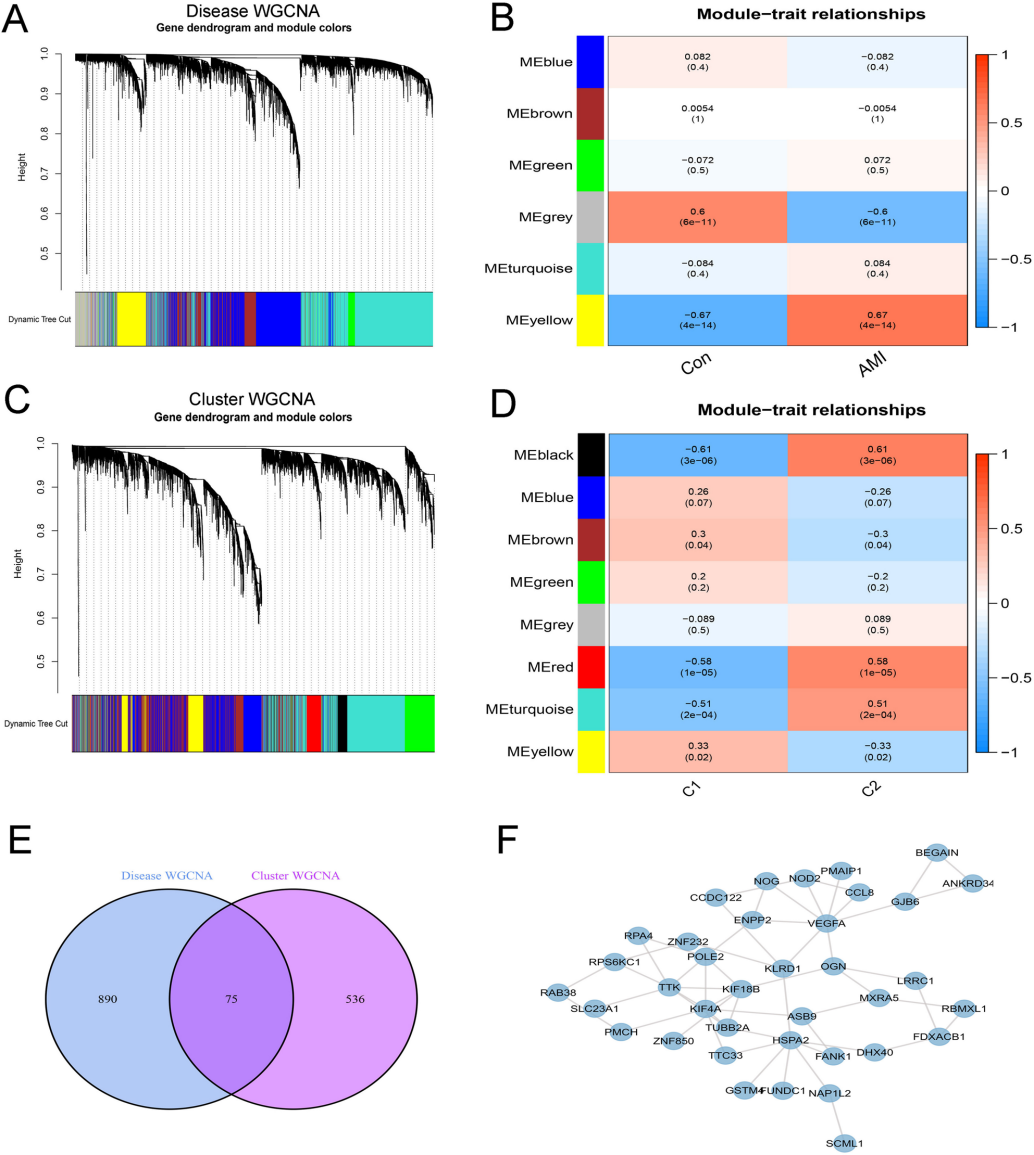
Disease and classification weighted gene co-expression network analysis (WGCNA) and protein-protein interaction (PPI) analysis. (**A**) Disease-related WGCNA; (**B**) Correlation of different modules with disease; (**C**) Classification-related WGCNA; (**D**) Correlation of different modules with classification; (**E**) Venn diagram of genes within key modules; (**F**) PPI analysis results

In the disease WGCNA analysis (Fig. 5A, B), six modules were identified, with two modules (yellow and grey) showing the strongest correlation with the disease. These modules contained a total of 965 genes. In the typing WGCNA analysis (Fig. 5C, D), eight modules were identified, and two modules (black and red) showed the strongest correlation with CRG subtyping, containing 611 genes. Intersecting the gene sets from both analyses resulted in 75 overlapping genes (Fig. 5E). These intersecting genes were further analyzed for PPI, with the resulting network displayed in Fig. 5F.

### Machine learning for screening key disease genes

**Fig. 6:**
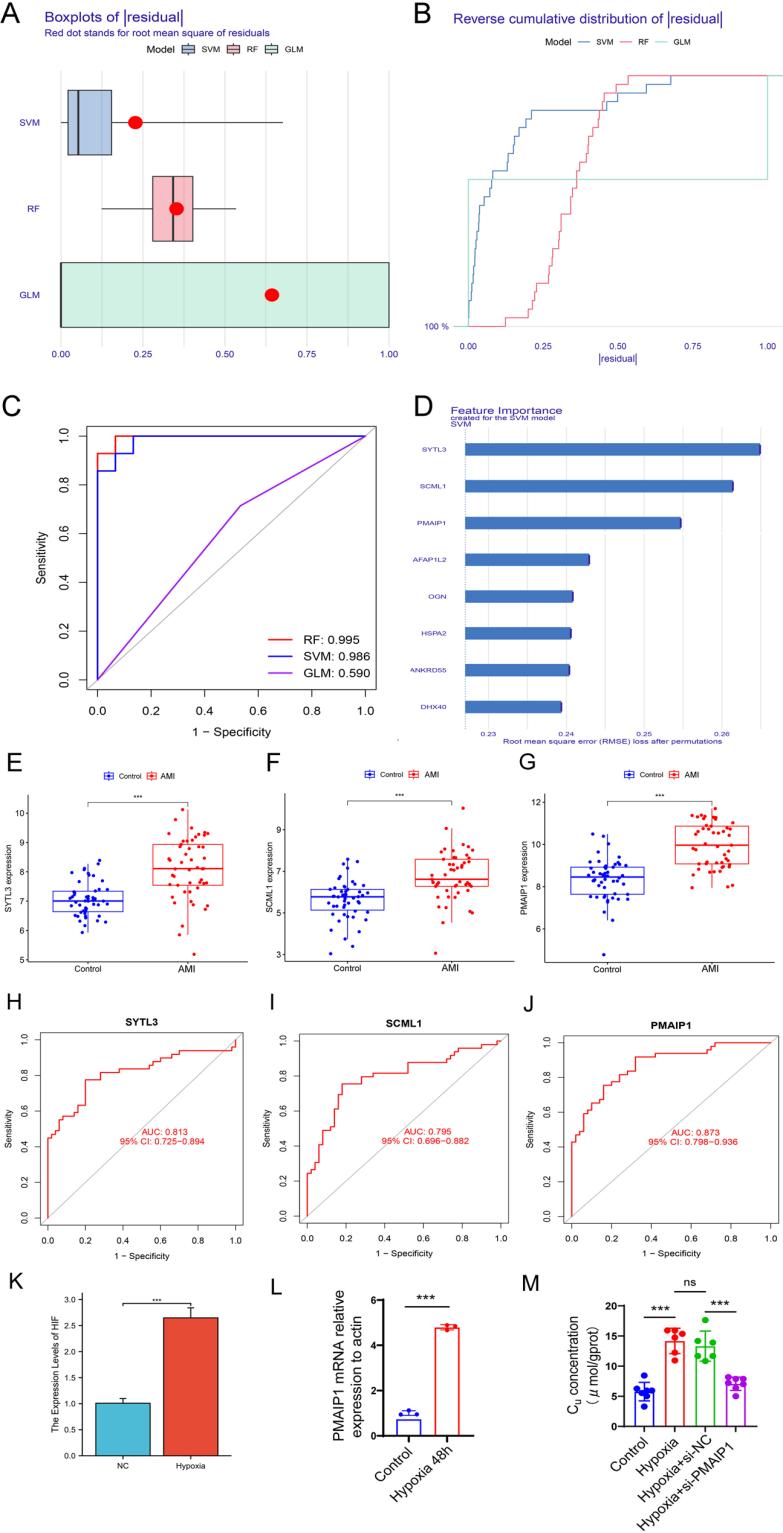
Identification, validation, and functional characterization of key disease genes. (**A**) Boxplot of residuals; (**B**) Reverse cumulative distribution of residuals; (**C**) Receiver operating characteristic curves for key gene screening; (**D**) Importance scoring of genes identified by Support Vector Machines (SVM). (**E**-**G**) Expression levels of key genes in disease versus control groups; (**H**-**J**) Receiver operating characteristic curves demonstrating the ability of key genes to differentiate between disease and healthy controls; (**K**) Expression levels of HIF-1α in hypoxia-induced HUVECs (exposed to 1% O₂ for 4 h) measured by RT-qPCR; (**L**) Expression levels of PMAIP1 in hypoxia-induced HUVECs measured by RT-qPCR; (**M**) Copper (Cu^2+^) levels assessed using the Cell Copper Colorimetric Assay Kit. ^***^p < 0.001. AMI, acute myocardial infarction; GLM, Generalized Linear Model; RF, Random Forest

Three machine learning methods—RF, SVM, and GLM—were employed to identify key disease-related genes. Based on the analysis of the residual boxplot, the reverse cumulative distribution plot, and the ROC curve, SVM was selected as the most effective method for screening key disease genes (Fig. 6A-C). The top three genes with the highest importance scores were identified as SYTL3, SCML1, and PMAIP1 (Fig. 6D).

### Key gene-transcription factor and key gene-small molecule compound interaction networks

The key gene-transcription factor interaction network analysis revealed that SYTL3 was linked to 23 transcription factors, SCML1 to 12 transcription factors, and PMAIP1 to 46 transcription factors (Supplemental Fig. 2A). Additionally, key gene-small molecule compound interaction network analysis showed that SYTL3 interacted with compounds such as aflatoxin B1, antirheumatic agents, and benzo(a)pyrene. SCML1 was associated with Zinc and vorinostat sodium selenite, while PMAIP1 was linked to copper sulfate, cyclosporine, genistein, and other small molecules (Supplemental Fig. 2B).

### Diagnostic ability of key disease genes for AMI

A comparison of the expression levels of key disease genes between the AMI group and the normal control group indicated that the expression levels of SYTL3, SCML1, and PMAIP1 were significantly higher in the AMI group (*p* < 0.05) (Fig. 6E-G). The AUC values of the ROC curves for these key genes were all above 0.79, confirming their strong diagnostic potential, with PMAIP1 demonstrating the highest capability to distinguish the AMI group from the healthy control group (Fig. 6H-J).

### PMAIP1 regulates intracellular copper levels in hypoxic endothelial cells

To validate hypoxic model efficacy, we quantified HIF-1α mRNA expression levels in HUVECs following hypoxic treatment. Quantitative PCR analysis demonstrated significant upregulation of HIF-1α mRNA expression in hypoxia-treated cells compared to normoxic controls (*p* < 0.001) (Fig. 6K). The above results suggested that PMAIP1 has excellent diagnostic value for AMI. Although PMAIP1 plays a critical role in tumor and immune-related cells, its function in AMI progression remains unclear [30]. To validate the role of PMAIP1 in endothelial cuproptosis, we first confirmed its expression under hypoxic conditions. RT-qPCR analysis demonstrated that PMAIP1 mRNA was significantly upregulated in HUVECs after 48 h of hypoxia exposure compared to normoxic controls (Fig. 6L). To further investigate the physiological function of PMAIP1, siRNA was used to knock down PMAIP1 expression in HUVECs. The efficiency of the knockdown was confirmed by RT-qPCR (Supplemental Fig. 3). Cu^2+^ levels were lower in the si-PMAIP1 + hypoxia group than in the si-NC + hypoxia group (Fig. 6M).

## Discussion

Cardiovascular diseases, particularly CAD, represent a significant global health burden. While advances in interventional therapies, including vascular revascularization techniques, have substantially improved outcomes for AMI patients, early diagnosis and precision-targeted therapeutic approaches remain critical for optimizing treatment efficacy. Given the limited understanding of cuproptosis—an emerging cell death mechanism—in cardiovascular pathophysiology, our study employed a comprehensive research strategy to systematically investigate the role of cuproptosis in AMI pathogenesis. Our investigation comprehensively characterized the relationship between CRGs and AMI through multilevel analyses. We identified nine differentially expressed CRGs and developed a robust diagnostic classification model incorporating six signature genes (AUC = 0.927). Additionally, we characterized two distinct molecular subtypes with unique biological profiles and identified key diagnostic genes, including PMAIP1, through integrated WGCNA and machine learning methodologies. This comprehensive investigation provides novel molecular targets and theoretical foundations for precision cardiovascular medicine.

Copper is an essential trace element in the body, playing vital roles in physiological processes such as mitochondrial respiration, antioxidant reactions, and the synthesis of biomacromolecules [31]. However, copper acts as a double-edged sword within cells; while it is a necessary cofactor for numerous enzymes, excessive copper can induce oxidative stress, ultimately leading to cell death. Both copper deficiency and overload can harm cells, prompting the body to maintain intracellular copper ion concentrations within a specific range via active internal balancing mechanisms. Previous studies have indicated that abnormal copper levels can lead to various cardiac conditions, including cardiac ischemia-reperfusion injury [32], arrhythmias [33], and cardiac hypertrophy [34]. In an observational study involving 41 AMI patients and healthy controls matched for age and sex, serum copper concentrations were significantly higher in AMI patients than in the control group (138 vs. 98 pg/dl, *p* < 0.001) [12]. This suggests that copper ions may play a critical role in the genesis of AMI and could serve as a potential therapeutic target.

Cuproptosis, a newly identified form of cell death caused by copper overload, occurs when excess copper binds directly to lipoyl proteins in the mitochondrial TCA cycle. This interaction results in abnormal aggregation of lipoyl proteins and the loss of iron-sulfur clusters in the respiratory chain complex, ultimately leading to toxic protein stress responses and cell death. A recent prospective cohort study by Kunutsor et al. [13] found significant positive correlations between circulating copper levels and both BMI and total cholesterol, alongside a negative correlation with HDL cholesterol. Elevated copper levels were associated with an increased risk of cardiovascular disease, with each 1 standard deviation increase in serum copper correlating with an 8% increased risk of cardiovascular disease. Additionally, Li et al. [35] utilized metabolomics to analyze metabolite level changes in pig cardiomyocytes following copper exposure, revealing that seven metabolites were up-regulated while 37 were down-regulated. These changes predominantly involved glycerophospholipid metabolism and the extension and degradation of fatty acids, aligning with findings from numerous previous studies [36–38]. Higher serum copper levels may heighten the risk of atherosclerotic heart disease by influencing lipid metabolism, promoting low-density lipoprotein oxidation, and exacerbating inflammatory responses, thus accelerating atherosclerotic plaque formation [13].

In recent years, multiple studies have investigated the role of cuproptosis in cardiovascular diseases, revealing both similarities and differences compared to our findings. Regarding CRGs identification, Liu et al. demonstrated that GLS serves as an important diagnostic gene in AMI, which aligns with our identification of GLS among the differentially expressed CRGs [39]. However, their investigation focused exclusively on a single gene, whereas our systematic analysis identified 9 differentially expressed CRGs and established a comprehensive diagnostic classification model incorporating 6 signature genes. In contrast, Chen et al. identified SLC31A1, SLC31A2, and SOD1 as diagnostic biomarkers in atherosclerosis [40], while our study revealed downregulated expression of genes including ATP7B and LIPT1. These discrepancies likely reflect distinct pathological mechanisms underlying AMI versus chronic atherosclerosis. Importantly, multiple studies have consistently reported upregulated NLRP3 expression in cardiovascular diseases [41], which strongly corroborates our findings and further validates the central role of inflammatory responses in cuproptosis-mediated cardiovascular injury.

Our AMI diagnostic classification model is based on six key CRGs that collectively represent critical pathways in cardiovascular disease pathogenesis. These genes form an integrated regulatory network spanning copper homeostasis, oxidative stress response, inflammation, and mitochondrial metabolism. NFE2L2 functions as a master regulator of cellular antioxidant responses, maintaining redox homeostasis through activation of antioxidant response element-driven gene transcription [42]. Its upregulation in AMI pathophysiology likely represents a compensatory protective mechanism against oxidative stress induced by ischemia-reperfusion injury. NLRP3 inflammasome serves as a central component of innate immunity, with its activation in AMI promoting maturation and release of pro-inflammatory cytokines IL-1β and IL-18, thereby driving myocardial inflammation and pyroptosis [41]. The copper transport machinery is represented by ATP7B, a critical protein governing intracellular copper efflux whose dysfunction underlies copper metabolism disorders such as Wilson’s disease [43]. In cardiovascular pathophysiology, ATP7B downregulation may compromise cardiomyocyte copper homeostasis, increasing susceptibility to copper toxicity-mediated cellular damage. Mitochondrial metabolism is regulated through LIPT1 and DBT, both essential enzymes in the lipoylation process required for TCA cycle enzyme activity. The fundamental mechanism of cuproptosis involves aberrant copper binding to these lipoylated proteins, resulting in protein aggregation and iron-sulfur cluster destruction [8]. GLS, a key enzyme in cellular energy and nitrogen metabolism, plays a pivotal role during cellular stress conditions. Under ischemic-hypoxic conditions, cardiomyocyte metabolic reprogramming relies on alternative substrates including glutamine [44]. GLS downregulation may indicate compromised metabolic adaptability during AMI, potentially limiting cardiomyocyte survival under stress. Collectively, these six genes constitute an intricate regulatory network that reflects the complex pathophysiological landscape of AMI. Their combined expression profile not only captures the multifaceted nature of cuproptosis-mediated cardiac injury but also provides novel mechanistic insights for therapeutic intervention. This gene signature offers both a robust foundation for AMI risk stratification and promising targets for developing cuproptosis-targeted cardiovascular therapies.

While our CRGs-based AMI diagnostic classification model performed excellently on our current dataset, its applicability in diverse populations and clinical settings requires careful and prospective validation. Firstly, since our model was developed using gene expression data predominantly from European and American populations, its generalizability to other ethnic groups needs targeted validation and calibration. This is critical as different populations may exhibit significant variations in gene expression patterns, copper metabolism, and susceptibility to cardiovascular diseases. Secondly, given that AMI is a heterogeneous disease with diverse molecular mechanisms across its subtypes, our model’s applicability will require validation through large-scale, multi-center studies. From a clinical perspective, this model has the potential to be useful in several stages of patient care, including the rapid screening of suspected AMI patients, guiding interventional treatment decisions, and informing prognostic evaluation. However, as this is a preliminary study, our model must be integrated with existing clinical diagnostic standards and biomarker data to form a more comprehensive and reliable diagnostic system.

We identified two molecular subtypes associated with cuproptosis in AMI patients. These subtypes exhibited differences in pathways closely related to AMI, such as fibrinogen formation, heart morphogenesis, and apoptosis. This study enhances our understanding of the molecular characteristics and biological functions of different AMI subtypes. Through WGCNA and machine learning, we identified three key genes significantly associated with AMI: SYTL3, SCML1, and PMAIP1. SYTL3, or synaptotagmin-like 3, is located on chromosome 6q25.3. The transport of lipids between cells involves vesicles, and the protein encoded by SYTL3 plays a pivotal role in this vesicle transport [45–47]. Prior studies have reported that a single nucleotide polymorphism in SYTL3-SLC22A3 and its haplotype are associated with the risks of hyperlipidemia and CAD [48, 49].

SCML1, an 18 kb transcription factor and polycomb group (PcG) gene, contains eight exons [50]. Knockout of the PcG gene in mice leads to various abnormal phenotypes, including impaired hematopoietic function, neural crest defects, and cardiac developmental malformations [51, 52]. Nan et al. [53] found that SCML1 regulates the transcription of the humanin (HN) polypeptide family in lung cancer, which has high homology with the mitochondrial genome, particularly with HN2 and HN8 localized in mitochondria. Increased SCML1 expression can disrupt mitochondrial function by regulating transcription within the HN polypeptide family.

PMAIP1, which encodes NOXA, is a member of the BH3-only subfamily that promotes apoptosis within the B-cell lymphoma-2 (Bcl-2) gene family [54]. PMAIP1 induces apoptosis by targeting the prosurvival molecule Mcl-1 and can also function as a tumor suppressor gene, influencing cell proliferation and apoptosis via p53-dependent or p53-independent pathways [55, 56]. Moreover, overexpression of PMAIP1 can activate apoptosis effectors that oligomerize, leading to perforation of the mitochondrial outer membrane, cytochrome C release, and activation of the caspase pathway [57]. In recent years, PMAIP1’s role in vascular diseases has gained attention; it is highly expressed in human acute aortic dissection samples and may mediate mitochondrial-dependent apoptosis in human aortic vascular smooth muscle cells by regulating Bax and Bcl-2 expression [58]. PMAIP1 expression has been found to increase in both transiently ischemic and ischemia-reperfused hearts [59–61]. Notably, Liu et al. [62] reported that PMAIP1 could serve as a valuable biomarker for early detection of AMI, with high expression correlating with increased inflammatory responses and immune cell infiltration.

In our study, we found that PMAIP1 expression is significantly upregulated under hypoxic conditions, suggesting it may be an early cellular response to ischemic stress. Our observation that PMAIP1 knockdown significantly reduced copper ion levels directly confirms its critical role in copper homeostasis. Given that PMAIP1 is a well-recognized pro-apoptotic member of the Bcl-2 family primarily localized to the mitochondria, its influence on cuproptosis likely stems from a profound impact on mitochondrial metabolic flux. We speculate that PMAIP1 may modulate the stability or activity of key enzymes within the TCA cycle, such as DLAT. In the context of copper accumulation, such interactions could exacerbate the aggregation of lipoylated proteins, a hallmark of cuproptosis that leads to proteotoxic stress and the loss of mitochondrial iron-sulfur cluster proteins. Therefore, the upregulation of PMAIP1 under hypoxia may predispose endothelial cells to copper-induced metabolic collapse by disrupting the delicate balance between mitochondrial respiration and copper detoxification pathways.

Our study presents several distinctive advantages. First, this study represents the investigation to integrate molecular subtyping with CRGs in AMI research, successfully identifying two molecular subtypes with distinct biological characteristics, whereas previous studies have primarily focused on differential gene identification alone. Second, we employed an integrated approach combining WGCNA with multiple machine learning algorithms for key gene selection, providing enhanced reliability compared to single-method approaches. Furthermore, we experimentally validated the critical role of PMAIP1 in endothelial cell cuproptosis through in vitro studies, thereby strengthening the translational relevance of our computational findings.

Our research findings provide significant insights for precision medicine applications in AMI. First, the diagnostic classification model based on six characteristic CRGs demonstrates excellent predictive performance, offering a novel tool for AMI risk stratification. This model can be seamlessly integrated into existing clinical risk assessment frameworks, providing clinicians with molecular-level evidence to develop individualized prevention strategies. The key genes we identified, including PMAIP1, SYTL3, and SCML1, exhibit substantial diagnostic value. Notably, PMAIP1 demonstrates exceptional diagnostic performance (AUC = 0.873), surpassing several traditional biomarkers and showing considerable potential for development as a novel circulating biomarker. Regarding therapeutic applications, PMAIP1 represents a critical regulatory gene in cuproptosis, with its expression levels directly influencing copper ion accumulation in endothelial cells. This mechanism provides a strong theoretical foundation for developing PMAIP1-targeted therapeutic strategies. Furthermore, upstream regulatory factors including NFE2L2 and NLRP3 may serve as promising therapeutic targets, potentially protecting endothelial cells through activation of antioxidant pathways or inhibition of inflammatory responses. The identification of distinct molecular subtypes further reinforces the clinical potential of precision medicine approaches. The two cuproptosis-related molecular subtypes we characterized exhibit significant differences in biological functions and pathway enrichment patterns, suggesting that patients with different subtypes may require tailored therapeutic strategies. However, translating these discoveries into clinical practice necessitates validation through large-scale prospective cohort studies, including comprehensive evaluation of the clinical utility of these genetic markers and assessment of the safety and efficacy of proposed therapeutic targets.

This study provides novel insights into the molecular biological features of cuproptosis in AMI. However, several limitations should be acknowledged. First, our dataset lacks detailed clinical information. This restricts our ability to analyze the relationship between different molecular subtypes and clinical outcomes, and specifically, to assess the correlation between the identified biomarkers and patient prognosis, treatment response, and complication rates. This is a major obstacle to advancing clinical translational research. Secondly, our bioinformatics analysis was based on a single GEO dataset derived from a European and American population. This reliance on a single dataset makes the findings susceptible to batch effects, platform-specific biases, and population-specific genetic variations, which may limit their generalizability to a broader patient population. While the sample size was adequate for our bioinformatics analysis, it remains relatively limited for robust biomarker discovery. Therefore, future research should focus on multi-center validation studies, larger sample sizes, detailed clinical correlation analyses, and comprehensive functional characterization of the identified pathways to fully realize the clinical potential of cuproptosis-targeted interventions in AMI management. Thirdly, because our dataset was derived from circulating endothelial cells of AMI patients, we chose HUVEC cells instead of cardiomyocytes for PMAIP1 functional validation. While endothelial dysfunction is a critical component of AMI pathophysiology, our approach limits a direct understanding of PMAIP1’s role in cardiomyocytes. Given that cardiomyocytes possess distinct metabolic characteristics from endothelial cells, the HUVEC model cannot fully recapitulate the unique physiological and pathological responses of cardiomyocytes in an ischemic-hypoxic environment. Finally, while we validated the function of PMAIP1 through in vitro experiments, our findings lack validation in animal models and require further mechanistic studies. This limits our understanding of its role in a whole-organism context.

## Conclusions

This study demonstrates the pivotal role of cuproptosis in AMI pathogenesis and identifies PMAIP1 as a potential central mediator of endothelial cell cuproptosis under hypoxic conditions. Our findings provide novel mechanistic insights into AMI and establish PMAIP1 as a promising diagnostic and therapeutic target for AMI management. Future investigations should focus on comprehensive mechanistic studies to further elucidate PMAIP1’s role in AMI pathogenesis and evaluate its therapeutic potential, while multicenter validation studies are essential to confirm the clinical translational significance of these discoveries.

## Supplementary Information

Supplementary Material 1.

Supplementary Material 2.

## Data Availability

The datasets used and analyzed in this study are available from the corresponding author upon reasonable request. All R analysis code has been provided as supplementary material.

## Abbreviations

AMI: Acute Myocardial Infarction
ATP: Adenosine Triphosphate
AUC: Area Under the Curve
Bcl-2: B-cell Lymphoma-2
BH3: Bcl-2 Homology 3
BMI: Body Mass Index
CAD: Coronary Artery Disease
cDNA: Complementary DNA
CDF: Cumulative Distribution Function
CRGs: Cuproptosis-Related Genes
DEGs: Differentially Expressed Genes
DMEM: Dulbecco’s Modified Eagle’s Medium
FBS: Fetal Bovine Serum
FDR: False Discovery Rate
GEO: Gene Expression Omnibus
GLM: Generalized Linear Model
GO: Gene Ontology
GSEA: Gene Set Enrichment Analysis
GSVA: Gene Set Variation Analysis
HN: Humanin
HUVEC: Human Umbilical Vein Endothelial Cells
KEGG: Kyoto Encyclopedia of Genes and Genomes
NC: Negative Control
PCA: Principal Component Analysis
PcG: Polycomb Group
PMAIP1: P53 Upregulated Modulator of Apoptosis 1
PPI: Protein-Protein Interaction
RF: Random Forest
ROC: Receiver Operating Characteristic
RT-qPCR: Real-time Quantitative Polymerase Chain Reaction
SCML1: SCM Polycomb Group Protein Like 1
SEM: Standard Error of the Mean
siRNA: Small Interfering RNA
STRING: Search Tool for the Retrieval of Interacting Genes
SVM: Support Vector Machines
SYTL3: Synaptotagmin-like 3
TCA: Tricarboxylic Acid
WGCNA: Weighted Gene Co-expression Network Analysis
