# Supplementary material for: Cuproptosis of endothelial cells in a hypoxic environment: from molecular classification to PMAIP1-targeted intervention
Source: Hereditas. 2026 Mar 19;163:55. doi: 10.1186/s41065-026-00667-w (PMC13123213; doi:10.1186/s41065-026-00667-w)
Supplement: Supplementary file 1 — Supplementary Material 1. [file 41065_2026_667_MOESM1_ESM.docx]

**Supplemental material**

**
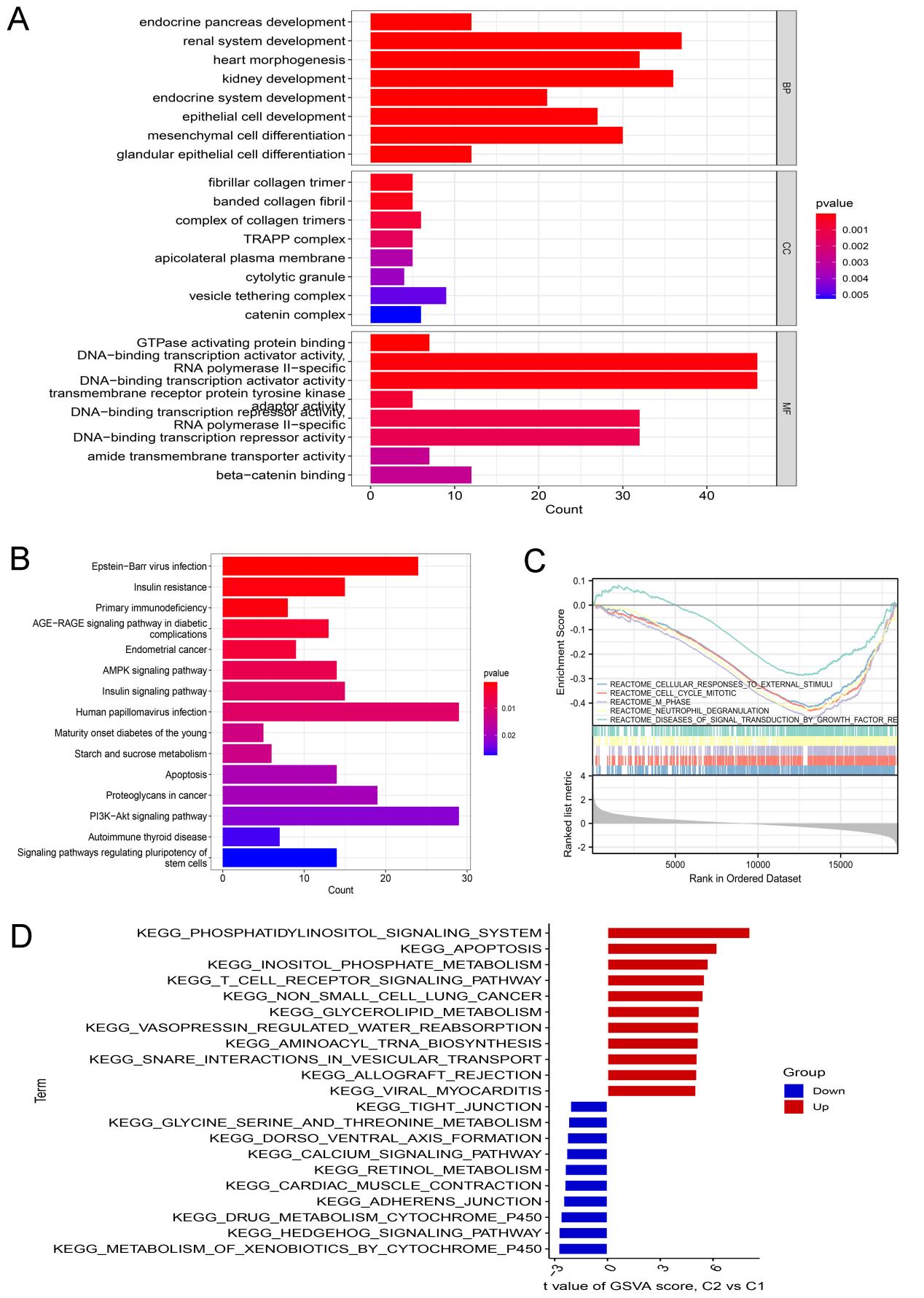
**

**Supplemental Fig. 1.** Functional enrichment analysis of the DEGs between the subtypes. (A) GO analysis of the DEGs between the subtypes; (B) KEGG enrichment analysis of the DEGs between the subtypes; (C) GSEA result of the DEGs between the subtypes; (D) GSVA result of the DEGs between the subtypes.


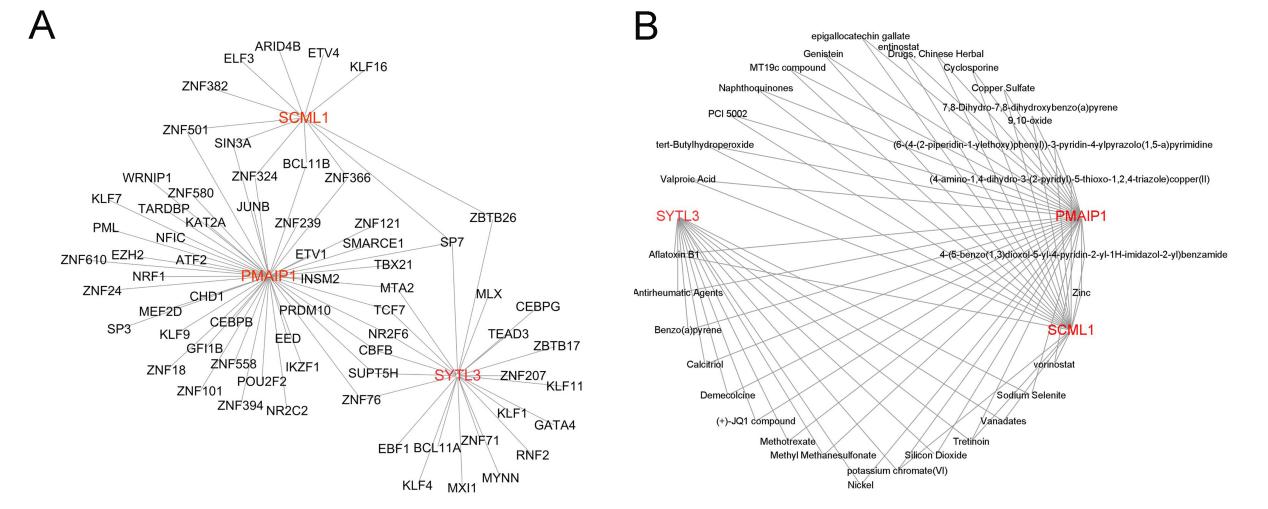


**Supplemental Fig. 2.** Key gene-transcription factor and key gene-small molecule compound interaction networks. (A) Interaction network diagram between key genes and transcription factors; (B) Interaction network diagram between key genes and small molecule compounds.


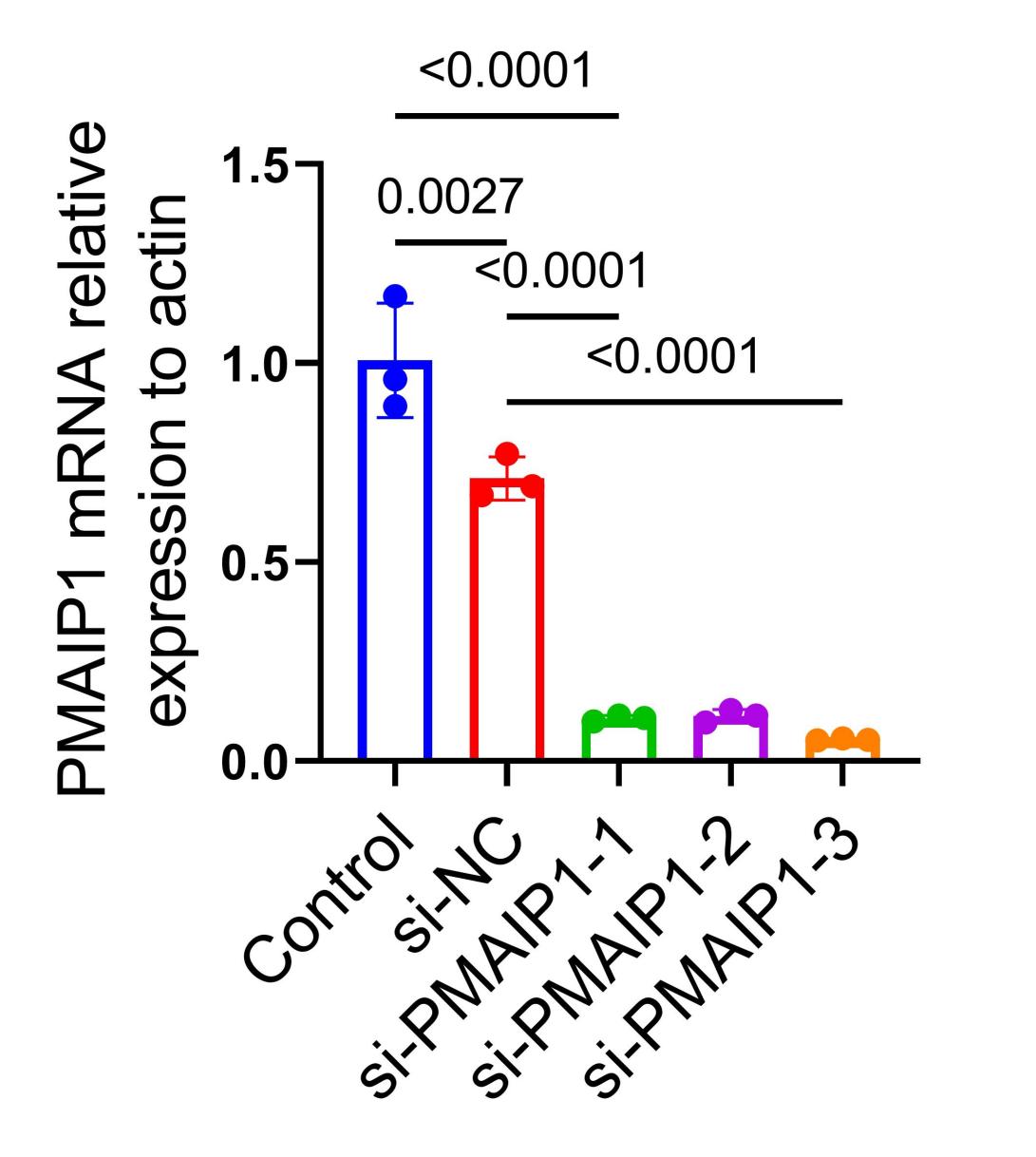


**Supplemental Fig. 3.** The efficiency of PMAIP1 knockdown was validated through RT-qPCR. (A) Interaction network diagram between key genes and transcription factors; (B) Interaction network diagram between key genes and small molecule compounds.

**Supplemental Table 1.** The target interference sequences of PMAIP1.

|  | Sense (5'→3') | Antisense (5'→3') |
| --- | --- | --- |
| si-PMAIP1-1 | CCGGCAGAAACUUCUGAAUTT | AUUCAGAAGUUUCUGCCGGTT |
| si-PMAIP1-2 | UACUCAACUCAGGAGAUUUTT | AAAUCUCCUGAGUUGAGUAGC |
| si-PMAIP1-3 | GGGAUGAGAGAACAUUAUATT | UAUAAUGUUCUCUCAUCCCAA |

**Supplemental Table 2.** The primer pairs used in RT-qPCR.

| Primer name | Forward（5'-3'） | Reverse（5'-3'） |
| --- | --- | --- |
| PMAIP1 | AGCTGGAAGTCGAGTGTGCTA | AGGTTCCTGAGCAGAAGAGTTT |
| SYTL3 | AAGACCTACCTGTTGCCCGA | CAGATGCCACACCGAGACC |
| SCML1 | TGATGCTCTGCAAAACCTGG | TTTGTCCACAGGGATCTCGC |
| HIF-1α | GTCTCCATTACCCACCGCTG | TTCATCAGTGGTGGCAGTGG |
| GAPDH | AGCCACATCGCTCAGACAC | GCCCAATACGACCAAATCC |
